# The impact of extracellular matrix proteins on bovine fibro‐adipogenic progenitor cell adhesion, proliferation, and differentiation in vitro

**DOI:** 10.14814/phy2.70283

**Published:** 2025-05-01

**Authors:** Perri Gish, Madison Stewart, Brandon Khuu, Nathaniel Meyer, Payam Vahmani, Lucas Smith

**Affiliations:** ^1^ Department of Neurobiology, Physiology, & Behavior, College of Biological Sciences University of California Davis California USA; ^2^ Department of Animal Science College of Agricultural and Environmental Sciences, University of California, Davis California USA; ^3^ Department of Physical Medicine and Rehabilitation School of Medicine, University of California, Davis California USA

**Keywords:** bovine, extracellular matrix, fibro‐adipogenic progenitor, fibronectin, mesenchymal stem cell

## Abstract

Fibro‐adipogenic progenitor cells (FAPs) are mesenchymal stem cells that produce extracellular matrix (ECM) and intramuscular adipocytes in skeletal muscle. While FAPs have demonstrated responsiveness to their physical environment, there is limited knowledge of how the ECM substrate of FAPs impacts their differentiation, particularly in livestock animals. We hypothesized that the ECM substrate FAPs are cultured on will differentially impact their adherence, proliferation, and differentiation. Through an initial screen of 9 ECM proteins and their combinations, significant variation of bovine FAP attachment and differentiation across coatings was observed. The ECM substrates fibronectin, collagen 6, vitronectin, and a combination of fibronectin and collagen 6 were selected for further testing. Notably, fibronectin increased cell proliferation and attachment rates, without impairing FAP adipogenic or fibrogenic differentiation compared to the other coatings. Benefits of fibronectin were maintained at lower concentrations and when combined with less favorable coatings such as collagen 6. When assessed for their adipogenic potential on each coating at different substrate stiffnesses, lipid accumulation decreased with increasing substrate stiffness, while cell attachment increased on stiffer substrates. Overall, these results demonstrate the high responsiveness of FAPs to their ECM substrate, along with highlighting fibronectin as a preferred substrate for in vitro experiments with bovine FAPs.

## 1 INTRODUCTION

Fibro‐adipogenic progenitor (FAP) cells are a mesenchymal‐like cell population residing in the interstitial space of skeletal muscle (Uezumi et al., 2010). Within skeletal muscle, FAPs can activate into myofibroblasts, which both deposit extracellular matrix (ECM) or they can differentiate into adipocytes, which create sites for later accumulation of intramuscular fat (Molina et al., 2021). In vivo, FAPs and those activated into myofibroblasts are the main cell types contributing to ECM deposition (Chapman et al., 2017; Negroni et al., 2022). The ECM is a dynamic, three‐dimensional web of proteoglycans and various fibrous proteins including fibronectin, laminin, and different types of collagens (Csapo et al., 2020; Frantz et al., 2010). In both muscle and fat homeostasis, the ECM helps maintain tissue structure and supports cell physiology such as proliferation, migration, and differentiation (Chen et al., 2023; Zhang et al., 2021). In vitro, FAPs (Loomis et al., 2022), adipose stem cells (Young et al., 2013), and preadipocytes (Takata et al., 2020) have been shown to be responsive to substrate stiffness. Additionally, the composition of cell culture substrates has been shown to modulate cell differentiation and attachment (Hausman et al., 1996; Shaheen et al., 2017; Vaz et al., 2012). Overall, in vitro experiments have demonstrated how different compositional and mechanical aspects of the ECM influence cell behavior, highlighting the importance of understanding the ECM's impact on a cellular level.

FAPs have largely been investigated for their role in muscle and ECM regeneration in homeostasis, and their contribution to fatty infiltration and fibrosis in diseased states (Contreras et al., 2021). Recently, FAPs have also gathered interest in agricultural research for their role in meat quality, particularly in beef research. Intramuscular fat in cattle (marbling) is a key factor in many beef grading systems around the world, including the United States, Korea, and Japan (Agricultural Marketing Service, n.d.; Baik et al., 2023). Higher marbling is typically associated with more flavorful, tender, and higher value meat; conversely, excess connective tissue is associated with a tougher meat product and overall lower meat quality. To increase meat quality, agricultural research is concerned with both driving FAPs towards adipogenic differentiation as well as improving rates of proliferation to increase intramuscular fat and create more sites for fat deposition within muscle (Maciel et al., 2022). Additionally, FAPs have grown in interest as a cellular source of fat (Dohmen et al., 2022) and structure (Ahmad et al., 2021) in the emerging field of cultivated meat, which aims to utilize tissue engineering techniques and cultured animal cells to create meat products for consumption. It is therefore critical to better understand how FAP cell physiology changes dependent on the ECM in which they reside.

Despite the interest in bovine FAPs for agricultural and food science research, there are still significant knowledge gaps in the factors driving FAP activation into myofibroblasts or differentiation into adipocytes. Furthermore, the establishment of optimized protocols for their use in vitro from livestock animals will be required to standardize their use. With an understanding of FAPs in vivo location, connection to the ECM, and the importance of substrate in cell culture, our objective was to better understand the impact of ECM molecules on bovine FAP behavior and improve their cell culture conditions. We hypothesized that ECM molecules as cell culture coatings would differentially impact bovine FAP attachment, proliferation, and differentiation. To address this question, we screened 36 different ECM molecule combinations for FAP attachment and differentiation and selected fibronectin, collagen 6, vitronectin, and a combination of fibronectin + collagen 6 for further testing. On these selected coatings, we measured proliferation, the timeline of cell attachment, adipogenic and fibrogenic differentiation, responses to media type, and responses to substrate stiffness. Our results demonstrate fibronectin increased FAP attachment rates and proliferation but do not impact FAP differentiation, supporting its use to enhance efficiency during in vitro experiments. Additionally, we found FAPs differed in attachment and adipogenic differentiation potential on varying substrate stiffnesses, demonstrating the need for further investigation into their mechanosensitive behaviors. Collectively, the present study provides the first in‐depth assessment of bovine FAP‐substrate interactions and highlights the need for further research on FAPs and their environment in livestock systems.

## 2 MATERIALS AND METHODS

### 2.1 FAP isolation

**FIGURE 1:**
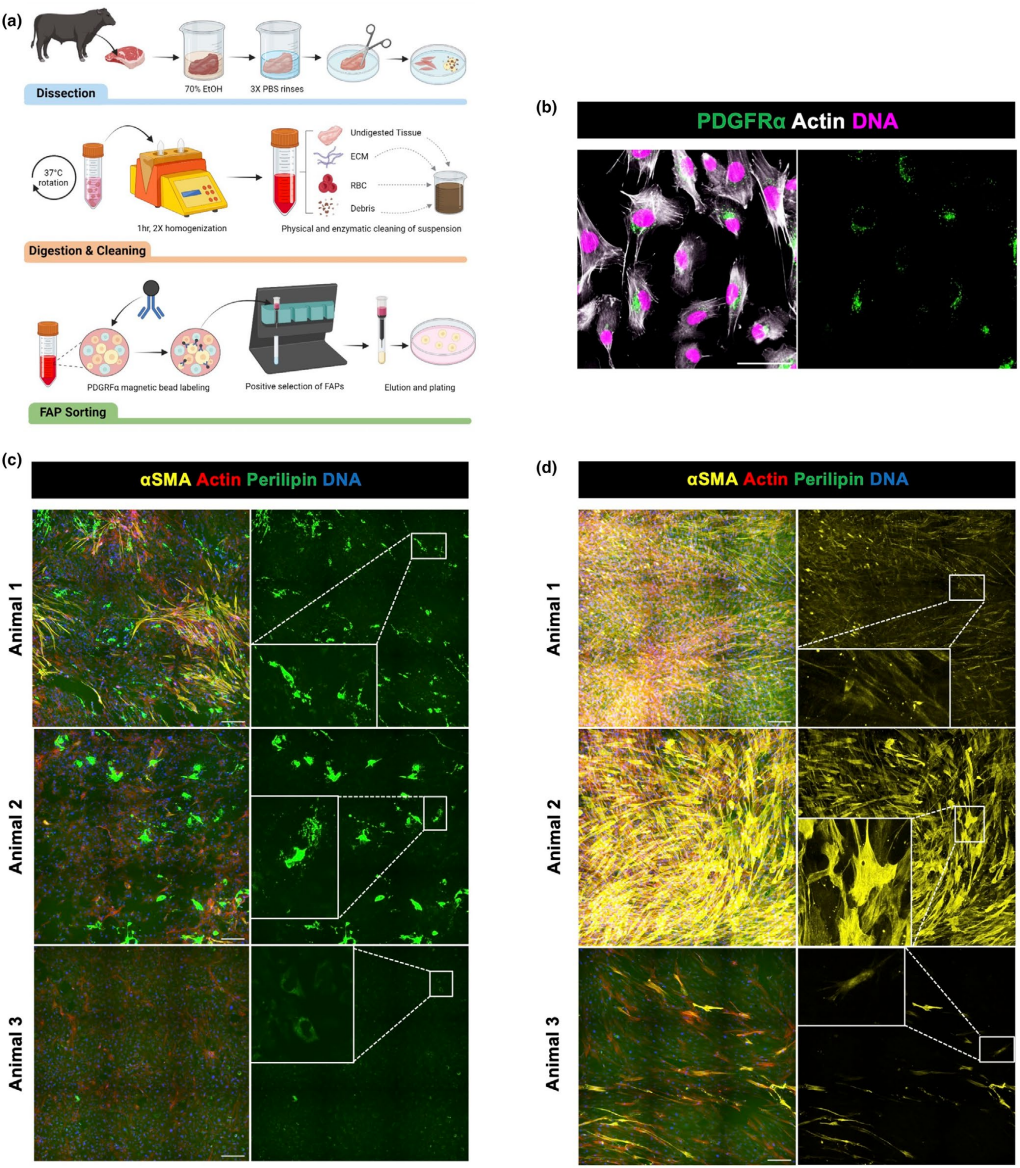
Bovine FAP isolation and verification. (a) Schematic of primary cell isolation protocol and FAP sorting using MACS. (b) Composite and single channel image of PDGFRα signal on animal 1 bovine FAPs. (c) Perilipin expression in FAPs after 10 days of adipogenic differentiation. (d) αSMA expression in FAPs after 10 days of fibrogenic differentiation. All cells plated on fibronectin (b–d). Scale bars are 50 μm (b) and 200 μm (c, d).

A schematic depicting our isolation procedure is included in Figure 1; this protocol was adapted and adjusted from those previously reported (Ding et al., 2018; Johnson et al., 2023; Loomis et al., 2022). Animals were not covered under an experimental IACUC protocol as tissue was collected based on availability post slaughter under USDA inspection at the UC Davis Meat Lab. Bovine skeletal muscle samples were acquired within 30 min after slaughter, and details on animals sampled are recorded in Table 1. Tissue was stored on ice in an antibacterial solution containing 1% penicillin streptomycin (Fisher 15140122), 0.5% gentamicin (Millipore Sigma G1272), and 0.4% amphotericin B (Fisher 15290018) for transport to the laboratory, where the rest of the isolation occurred in a biosafety cabinet. Muscle samples were sterilized in 70% ethanol for 5 min, followed by three 5‐min rinses in 1× PBS. Samples were then further dissected to remove connective tissue and subcutaneous fat, weighed into 1–2 g portions, minced into 0.5 cm or smaller pieces, and then suspended in a 2000 units/mL type II collagenase (Fisher 17101015) solution with 2% penicillin streptomycin in warmed DMEM (Ding et al., 2018). The digestion was rotated at 37°C for 1 h, with mechanical homogenization at 30 and 60 min via the GentleMACS Dissociator on the “m_muscle_01” setting (Miltenyi Biotech). Following the last homogenization, cells in suspension were removed from undigested tissue via alternating cycles of centrifugation at 70g to pellet tissue and 500g to pellet cells. Collected cell suspensions from animals 1–3 were passed through a 100 μm filter. Additionally, animals 2 and 3 were treated with a debris removal solution according to the manufacturer's instructions (Miltenyi Biotech 130‐109‐398). Erythrocytes were then removed by incubating the cell pellet in a 1× ACK solution (Ding et al., 2018) for 1 min and then re‐pelleted. The suspension was passed through a 40 μm filter, and cells were counted. FAPs were sorted from the cell suspension via magnetic activated cell sorting (MACS) as previously described (Loomis et al., 2022). Briefly, FC receptor binding was blocked using an FC blocking reagent, and then PDGFRα+ cells were labeled with CD140a microbeads (Miltenyi Biotech 130‐101‐502). The cell suspension was passed through a MS column (Miltenyi Biotech 130‐042‐201) attached to a magnetic separator allowing for positive selection of PDGFRα+ cells.

**TABLE 1:** Cattle used for cell isolations.

Cattle sample ID	Breed	Sex	Age at slaughter, months	Sample area
1	Angus	Heifer	15	Shank
2	Angus	Heifer	22	Brisket
3	Angus	Steer	18	Brisket

### 2.2 General cell culture

Cells were grown in BoGrow media (F‐10 media, Fisher 11550043) with 20% fetal bovine serum (Biowest S1620), 1% penicillin/streptomycin (Fisher 15140122), 5 ng/mL FGF‐2 (Fisher PHG6015), 50 μg/mL gentamicin (Millipore Sigma G1272), and 1 μg/mL amphotericin B (Fisher 15290018) and given fresh media every 2–3 days (Ding et al., 2018). Stocks of cells were frozen overnight in fetal bovine serum with 10% dimethyl sulfoxide (Fisher BP2311) at −70°C in a CoolCell™ chamber (Fisher 07210004) and stored in liquid nitrogen.

### 2.3 Chip culture and design

**FIGURE 2:**
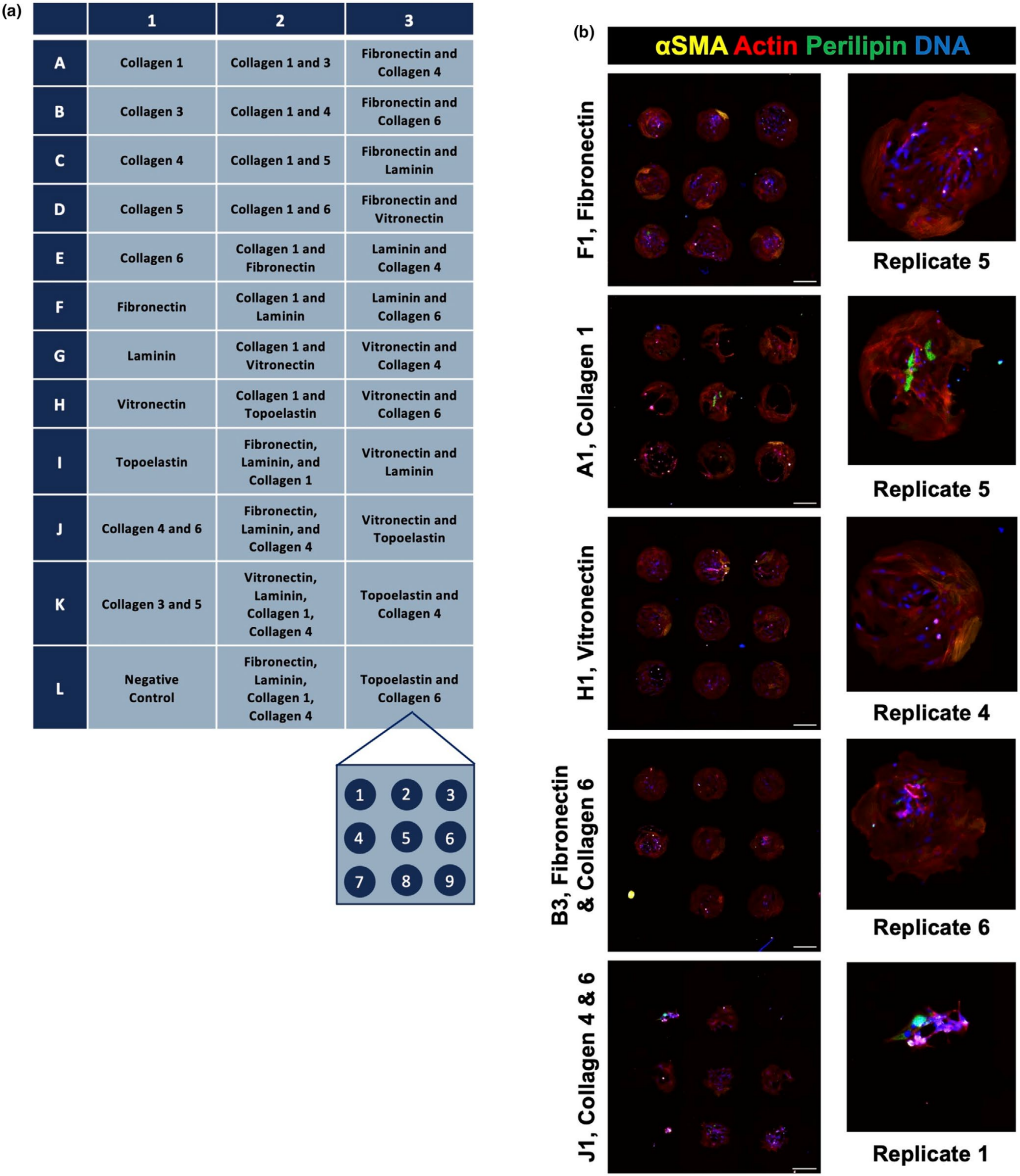
ECM Array Chip. (a) Advanced Biomatrix ECM Select Array Kit Ulta‐36 legend and coating replicate layout. (b) Representative immunofluorescent images showing Perilipin and αSMA signal after 5 days of differentiation in BoFat media. Chip cultured using P4 Animal 2 FAPs. Scale bars are 200 μm.

The initial coating screen was done using the ECM Array Kit Ultra‐36 chip (Advanced Biomatrix 5170) which contained 9 ECM components in 36 different combinations with 9 replicates per combination, each 200–400 μm in diameter; the layout of the chip is depicted in Figure 2. Animal 2 passage 4 FAPs were plated onto the ECM array chip according to manufacturer instructions. Briefly, 250,000 live cells in 5 mL of BoGrow were plated evenly over the array. The cells were allowed to attach and proliferate for 24 h at 37°C, before the media was changed to BoFat to induce adipogenic differentiation; BoFat is BoGrow with 0.25 μM dexamethasone (Fisher 1126100), 0.5 mM IBMX (VWR 102516252), 2.2 μg/mL troglitazone (Fisher 501150786), and 1 μg/mL insulin (Millipore Sigma 91077C) (Loomis et al., 2022). Cells were differentiated on the chip for 5 days, washed twice using Hank's Balanced Saline Solution (ThermoFisher AAJ67763AP), and finally fixed with 4% paraformaldehyde. 5 days of differentiation was used, as opposed to our standard 10, due to time in culture limitations of the chip. The chip was stored at 4°C in 1× PBS until immunofluorescent staining.

### 2.4 Coating procedure

All assays using the 5 selected coatings were plated at a 1.56 μg/cm^2^ coating density using 5 μg/mL solutions; this concentration was selected to standardize coatings while meeting manufacturer recommended concentrations for each coating individually. Coatings used include fibronectin (Human, Advanced Biomatrix 5050), vitronectin (Human, Fisher A14700), and collagen 6 (Bovine, Southern Biotech 1300‐02S). The collagen 6 + fibronectin coating was made using a 1:1 ratio of these stocks, matching the total protein coating density. Stock concentrations of each coating were thawed on ice in a biosafety cabinet and diluted to a 5 μg/mL solution in 1× PBS; the collagen 6 + fibronectin coating being each at 2.5 μg/mL. 100 μL of each solution was plated to each well on a TC treated plastic 96 well plate and on selected stiffnesses of a softwell 96 well plate (Matrigen Softwell 96 Glass with easy coat). Each plate was then rested for 1 h at ambient temperatures, washed 3 times with 1× PBS, and then covered with PBS and stored at 2–8°C until cells were plated.

### 2.5 Proliferation and differentiation assays

Proliferation assays were plated with 5000 live cells/well on each coating on a 96 well plate. Cells were cultured in BoGrow with 10 μM EdU (Fisher A10044) at 5% CO_2_ and 37°C for 24 h before fixing. For differentiation assays, 5000 live cells/well were plated on each coating in a 96 well plate and cultured in BoGrow for 2–3 days until 90% confluence was achieved. For the differentiation on different stiffnesses, a coated 96 softwell plate with polyacrylamide hydrogels was used on the 2 kPa, 8 kPa, and 25 kPa stiffnesses (Matrigen). For fibrogenic differentiation, at confluency cells were changed to fresh BoGrow and then cultured for 10 days, with media refreshed every 2–3 days. For adipogenic differentiation, upon confluency cells were switched to either BoFat media (BoGrow with 0.25 μM dexamethasone (Fisher 1126100), 0.5 mM IBMX (VWR 102516252), 2.2 μg/mL troglitazone (Fisher 501150786), and 1 μg/mL insulin (Millipore Sigma 91077C)) (Loomis et al., 2022) or FFA media (BoFat with 10 μM FFA extract) and cultured for 10 days with media changed every 2–3 days. Pure oleic acid (Nu‐Check Prep) was dissolved in 100% ethanol to yield a 100 mM stock for use in FFA media. To reduce forces applied to lipid‐accumulating cells, BoFat and FFA media were plated in excess of ~200–300 μL per well, and media was slowly hand‐aspirated using a p1000 pipette. Approximately 50–75 μL/well of old media was left during each media change, and new media was slowly dispensed using a p1000 pipette on the side of the well. Passage 3–5 FAPs were used for these assays, with cells of the same passage for each assay. All assays were fixed by incubating with 4% paraformaldehyde for 15 min and then stored at 2–8°C under PBS until staining.

### 2.6 Adhesion time course

Passage 5 cells from each animal were counted and suspended in BoGrow to create a stock solution. From this suspension, an equal number of live cells were plated on each coating and cultured in BoGrow for 1, 3, or 6 h; each time point was conducted on a separate plate, using 3 technical replicates. At the indicated time point, PFA was added to each well's culture media to reach a final concentration of 4% PFA and allowed to incubate for 15 min; no washing was performed between plating cells in each well and the start of fixation. After 15 min, plates were rinsed with PBS and stored at 2–8°C under PBS until staining.

### 2.7 Immunofluorescent staining, imaging, and image analysis

Fixed assays were retrieved from 2 to 8°C storage, permeabilized in a 0.5% triton (Fisher BP151‐500) solution for 10 min, and blocked in a 5% bovine serum albumin solution (BSA) (Fisher BP1600‐100) for 30 min. Samples were then incubated with primary antibodies in 5% BSA overnight at 2–8°C. The following day, samples were incubated with secondary antibodies and Acti‐Stain 555 Phalloidin (Fisher 50646254, 1:250) in 0.1% BSA for 90 min at ambient temperature. Lastly, nuclei were stained with Hoechst 33,342 (Fisher H3570, 1:2000) in 0.1% BSA for 15 min. Antibodies and stains used can be found in Table 2. Secondary staining, following washes, and storage until imaging were done protected from light. The ECM array chip was allowed to dry before imaging. Proliferation assays were stained as previously described (Flomerfelt & Gress, 2016) and visualized using a fluorescent azide. All incubations were done under gentle shaking on an orbital shaker. All fluorescent imaging was achieved using a DMi8 stand and dry 20× objective for 96 well assays, a 10× objective for ECM array chip, and a 63× objective for PDGFRα staining. All imaging used a Leica DFC9000 GTC camera and LAS X software. Standard exposures for each channel were used as follows: alexa 488/FITC 150 ms, hoechst/DAPI 50 ms, acti‐stain/rhodamine 30 ms, alexa 647/Cy5 100 ms. All differentiation and proliferation assays were imaged per condition at a standard size of 1830 μm by 1830 μm. PDGFRα images were imaged at a 200 μm by 200 μm (Figure 1b, Figure S3). Replicates in the ECM chip assay measured 200–400 μm in diameter. Images were analyzed in FIJI: ImageJ 1.53 t. For proliferation assays, a threshold was set to identify all Hoechst+ nuclei as well as EdU+ nuclei, and then nuclei were counted. For differentiation assays, the total Actin+, Perilipin+, and ⍺SMA area was measured in microns. Nuclei were also counted as described for Hoechst+ nuclei in the proliferation assay. All ImageJ scripts are available upon request from the corresponding author.

**TABLE 2:** Antibodies used for immunofluorescent staining.

Antigen	Host species	Vendor	Catalogue	Dilution
Perilipin‐1	Rabbit	Cell Signaling	9349s	1:200
αSMA	Mouse	Fisher	MS113P0	1:800
PDGFRα	Rabbit	Abcam	203491	1:75
Donkey anti‐Mouse IgG Alexa Fluor Plus 647	Donkey	Fisher	A32787	1:500
Donkey anti‐Rabbit IgG Alexa Fluor Plus 488	Donkey	Fisher	A32790	1:500

### 2.8 Statistical analysis

Statistical analysis was conducted using R (version 4.4.2) within RStudio (version 2024.12.0 + 467); lme4, emmeans, and magrittr packages were used (Bache & Wickham, 2014; Lenth, 2017). Experiments followed a randomized complete block design, with animal as the blocking factor. For each response variable, a linear mixed‐effects model was created, and overall treatment effects were analyzed using an ANOVA with Satterthwaite's method. ANOVA tests were followed by pairwise comparisons, when applicable, using the Kenward‐Roger degrees of freedom method and Tukey's method for *p*‐value adjustments. Statistical analysis was conducted in consultation with a statistician. Graphs were made in GraphPad Prism (version 10.3.1 (464)); graph bars represent the minimum, maximum, and mean value for each treatment. Only significant *p*‐values are depicted on graphs (*p* ≤ 0.05); *p*‐values were added by hand from analysis conducted in R.

## 3 RESULTS

### 3.1 Bovine FAPs can be isolated via MACS

Bovine FAPs were isolated from skeletal muscle samples from 3 Angus Cattle and sorted for PDGFRα+ cells using MACS (Table 1, Figure 1), the canonical surface marker of FAPs. The collected cell sample showed positive PDGFRα expression and a highly enriched population via immunofluorescent staining (Figure 1b). To further confirm cell identity, FAPs from each animal were adipogenically (Figure 1c) or fibrogenically (Figure 1d) differentiated for 10 days. Cells were stained for αSMA, a marker of myofibroblasts, and perilipin, marking lipid droplets in adipocytes. Positive expression for both lineages of FAPs was observed (Figure 1c,d).

### 3.2 FAPs preferentially attach and differentiate on different surface ECM coatings

**FIGURE 3:**
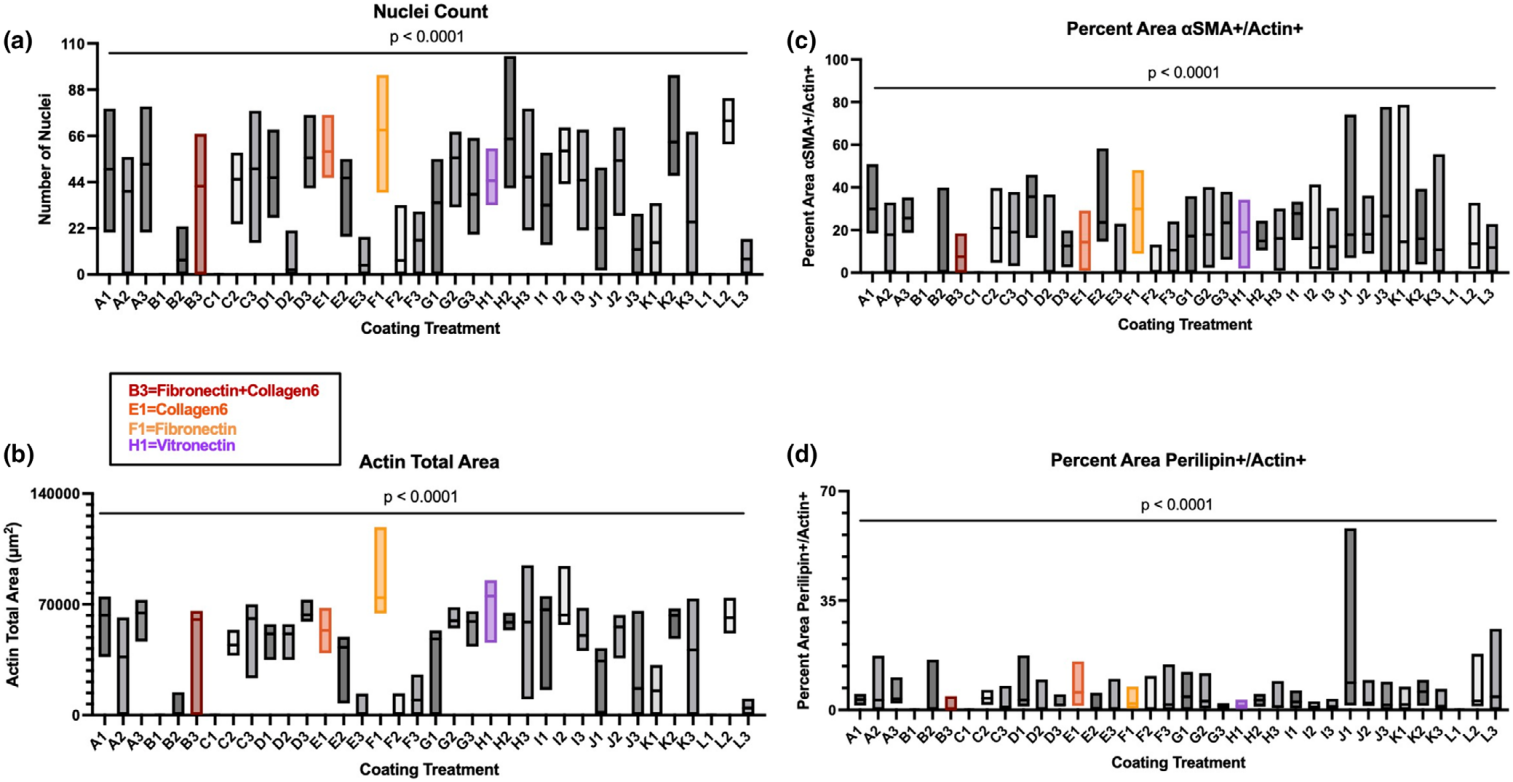
Quantification of FAP performance on the ECM Array Chip depicted in Figure 2. X‐axis values represent each coating treatment depicted in Figure 2a. (a) Quantification of total Hoechst+ cells. (b) Quantification of total Actin+ area. (c) Perilipin+ area as a percentage of the total Actin+ area. (d) αSMA+ area as a percentage of total Actin+ area. Coatings used in following select assays: B3 = Fibronectin + Collagen6, E1 = Collagen6, F1 = Fibronectin, H1 = Vitronectin. *n* = 9 replicates. Chip cultured using animal 2 FAPs.

To screen a broad spectrum of adhesion proteins for FAP attachment and differentiation, we plated and grew Animal 2 FAPs on an ECM Array Kit Ultra‐36 chip, which contained 9 different coatings plated individually and in different combinations; each coating treatment contained 9 replicates (Figure 2a). After 24 h, culture media was switched from BoGrow, growth media, to BoFat media with adipogenic inducers to stimulate adipogenic differentiation for 5 days. On day 6, the chip was fixed and stained to view αSMA, perilipin, nuclei, and actin (Figure 2b). Nuclei count and cell occupied area varied across coating treatments (*p* < 0.0001; Figure 3a,b). Select coatings, such as fibronectin, had high cell counts and cell occupied area; other coatings had poor cell attachment, such as collagen 1 and collagen 4 combinations, as well as collagens 1 or 4 in combination with laminin (Figure 3a,b). For FAP differentiation, markers of adipogenic (perilipin) and fibrotic (⍺SMA) differentiation were divided by the cell occupied (actin+) area to adjust for differences in initial cell attachment. Variation between coatings treatments for both measures was observed (*p* < 0.0001); in particular, coatings that promoted attachment also had higher relative myofibroblast area than others (Figure 3c). For adipogenic differentiation, significant variation was also observed, and collagen 6 was present in many coating treatments that had high relative lipid accumulation (Figure 3d). This result is consistent with existing literature that collagen 6 promotes adipocyte behavior and function (Oh et al., 2021; Pasarica et al., 2009). With regard to attachment, fibronectin supported the largest actin total area, high nuclei count, and has improved cell attachment in existing literature (Arredondo et al., 2021; Vaz et al., 2012; Williams et al., 2008); similarly, vitronectin promoted moderate to high cell attachment and cell number individually in this screen and is commonly used with embryonic stem cell culture (Heydarkhan‐Hagvall et al., 2012; Li et al., 2010; Yap et al., 2011). Based on the ECM protein interactions, individual effects, existing literature, and with consideration of experimental structure, we selected fibronectin, collagen 6, fibronectin + collagen6, and vitronectin for follow‐up experiments.

### 3.3 Fibronectin enhances FAP attachment and proliferation

**FIGURE 4:**
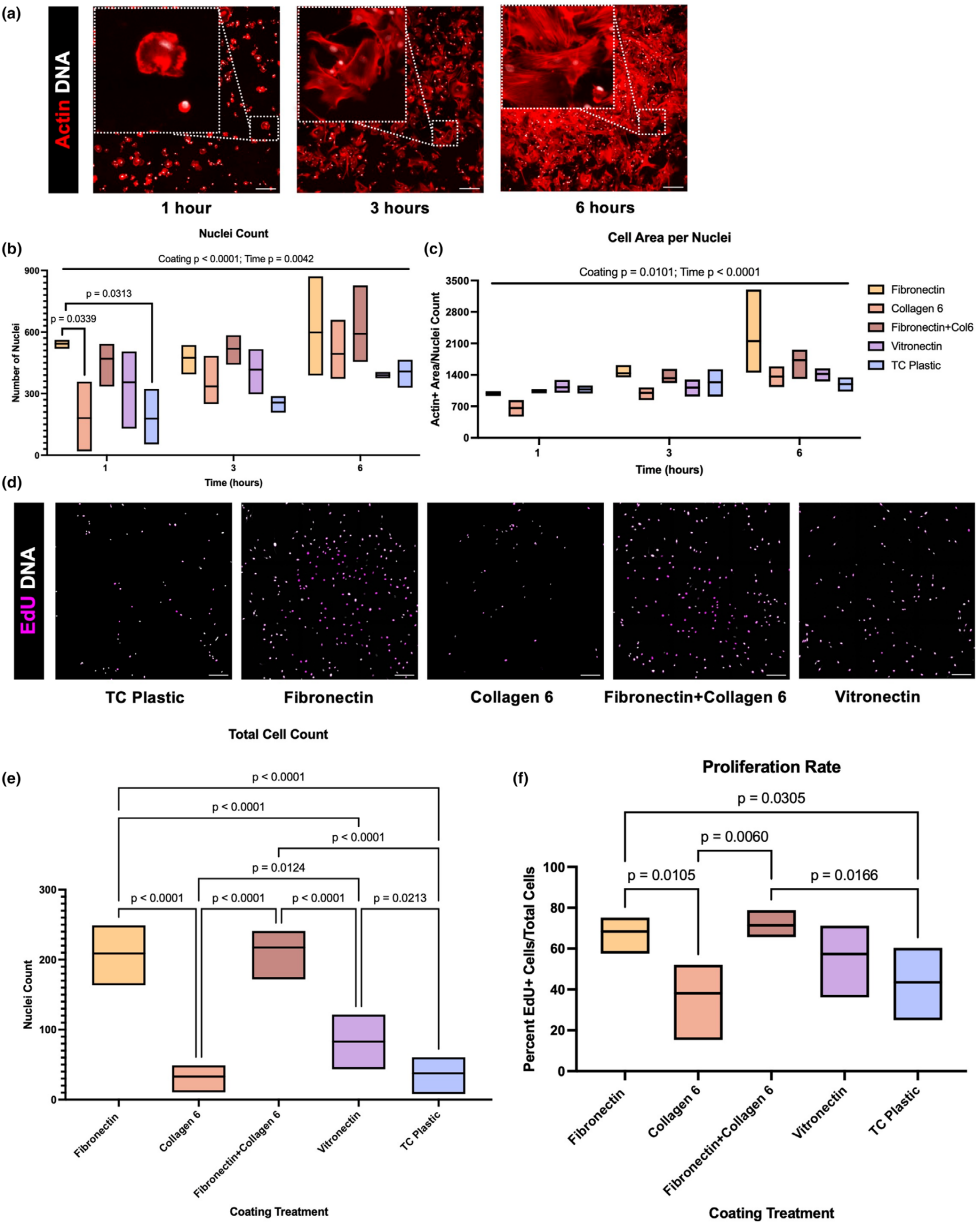
FAP attachment and proliferation on select substrates. (a) FAPs on fibronectin at 1, 3, and 6 h timepoints. (b) Quantification of total Hoechst+ FAPs for each coating at 1, 3, and 6 h. (c) Quantification of Actin+ area (μm2)/Hoechst+ FAPs for each coating at 1, 3, and 6 h. (d) FAPs after 24 h. treatment with EdU from Animal 2. (e) Quantification of total Hoechst+ cells 24 h. post‐seeding. (f) Quantification of EdU+/Hoechst+ FAPs 24 h post‐seeding and EdU treatment. *N* = 3 cattle. Scale bars are 200 μm.

To assess the four selected coatings' impact on cell adhesion, an adhesion time course was performed to further characterize the rate of cell attachment on each coating. To determine cell attachment at 1, 3, and 6 h, we measured the total number of attached cells (nuclei count) as well as measured the total attached cell area (actin+ area in μm^2^/nuceli count) (Figure 4a). The number of cells (*p* = 0.0042) and the cell area (*p* < 0.0001) increased with increasing time, and both measures were significantly impacted by coating (*p* < 0.0001 and *p* = 0.0101, respectively) (Figure 4b,c). While all coatings achieved a similar number of cells by 6 h, fibronectin attached more cells in the first hour compared to collagen 6 (*p* = 0.0339) and TC plastic (*p* = 0.0313) (Figure 4b).

To expand on these findings, we then sought to investigate each coating's impact on cell proliferation and attachment within 24 h via an EdU assay (Flomerfelt & Gress, 2016). Contrastingly to our finding that all coatings achieved similar numbers of cells after 6 h (Figure 4b), we found significant variation in cell attachment across coatings at 24 h (Figure 4e). Both fibronectin and fibronectin + collagen 6 had more nuclei at 24 h compared to all other coatings (*p* < 0.0001) and achieved 4.7‐fold more nuclei compared to TC plastic; both fibronectin coatings had similar numbers of nuclei (*p* = 0.9324) (Figure 4e). Vitronectin also had more nuclei than collagen 6 (*p* = 0.0124) and TC plastic (*p* = 0.0213) after 24 h, but still less than either fibronectin coating (*p* < 0.0001) (Figure 4e). Regarding proliferation rates, both fibronectin coatings had higher ratios of EdU+ cells/total cells compared to collagen 6 and TC plastic (*p* < 0.0305); additionally, vitronectin had similar proliferation rates to fibronectin (*p* = 0.4871) and fibronectin + collagen 6 (*p* = 0.2861), but vitronectin was also not significantly different from TC plastic (*p* = 0.2948) or collagen 6 (*p* = 0.0997) (Figure 4f). Overall, fibronectin coatings promoted faster attachment in the first hour after plating, more total cells at 24 h, and higher proliferation rates compared to TC plastic.

### 3.4 Media composition and substrate stiffness, but not coating treatment, drive FAP differentiation

**FIGURE 5:**
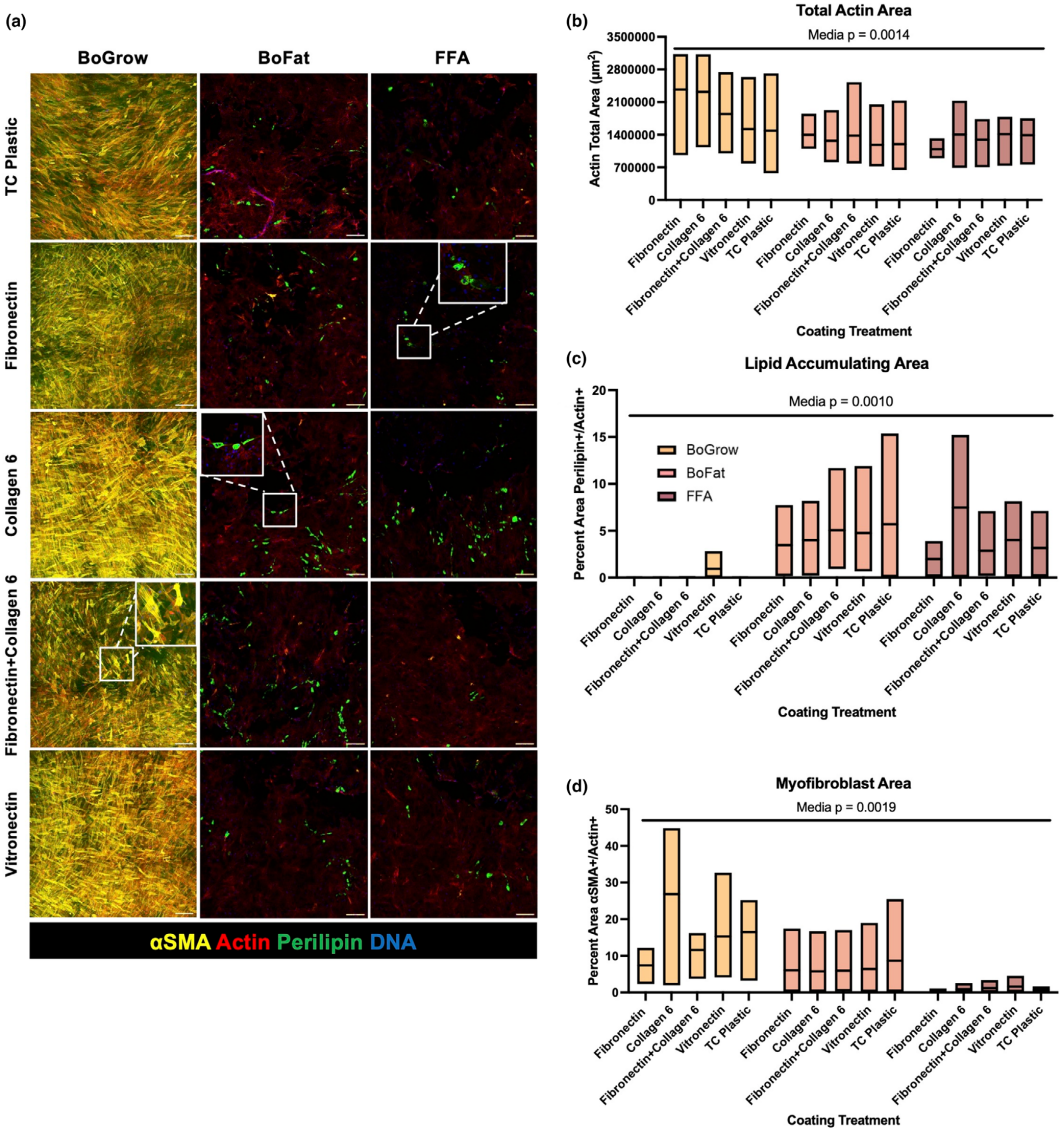
FAP differentiation across different substrates. (a) Immunofluorescent images of FAPs after 10 days of differentiation in the indicated media. (b) Quantification of total Actin+ area between coatings and media conditions. (c) Quantification of percent perilipin+/Actin+ area across media and coating conditions. (d) Quantification of percent αSMA+/Actin+ area between media and coating conditions. *N* = 3 cattle and *n* = 3 medias. Scale bars are 200 μm.

FAPs were adipogenically and fibrogenically differentiated on the selected coatings for 10 days to assess coating impact on FAP cell fate. Upon confluency, FAPs were left in BoGrow to prompt myofibroblast differentiation, or were switched to either BoFat or FFA media containing adipogenic inducers to prompt adipogenic differentiation (Loomis et al., 2022). FFA media contained supplemental oleic acid in BoFat media to investigate if fatty acids affected lipid accumulation of FAPs and their end‐stage attachment. Media type significantly impacted the total actin+ area (*p* = 0.0014), and non‐adipogenically differentiating cells in BoGrow trended to have more cell‐occupied (actin+) area (Figure 5b). Similarly, media type significantly impacted the relative lipid‐accumulating area (*p* = 0.0010) and relative myofibroblast area (*p* = 0.0019); BoFat and FFA media trended to have higher relative lipid‐accumulating areas than BoGrow, and BoGrow trended to have higher relative myofibroblast area compared to BoFat and FFA (Figure 5c,d). Coating treatments had no significant effect on end‐stage attachment (*p* = 0.6397), relative lipid‐accumulating area (*p* = 0.7158), nor relative myofibroblast area (*p* = 0.7208).

**FIGURE 6:**
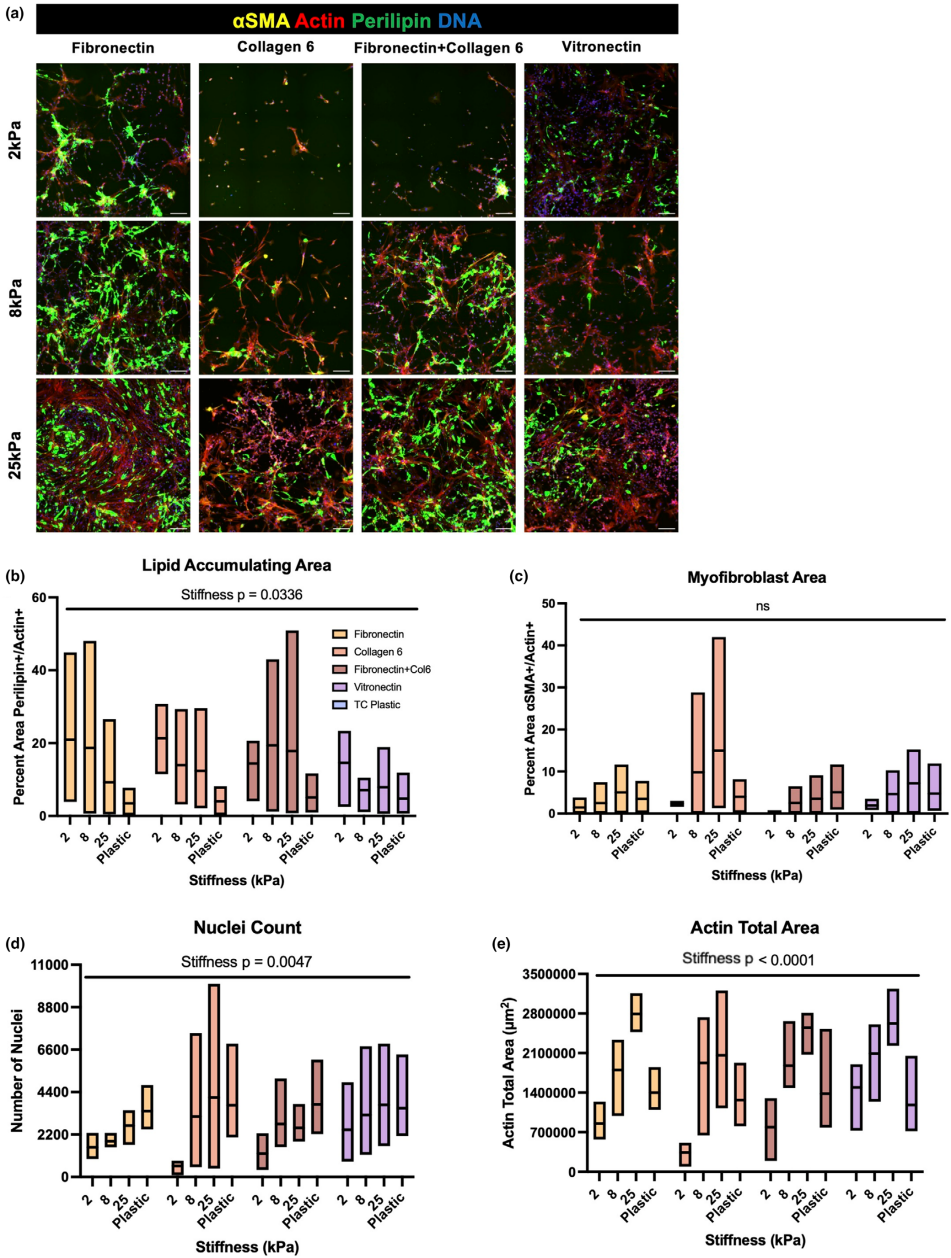
FAP differentiation on each coating at different stiffnesses. (a) Immunofluorescent images of FAPs after 10 days of differentiation on the indicated coating at 2, 8, or 25 kPa. (b) Quantification of percent Perilipin+/Actin+ area across coating types and stiffnesses. (c) Quantification of percent αSMA +/Actin+ area across coating types and stiffnesses. (d) Quantification of number of total Hoechst+ nuclei for each coating type and stiffness. (e) Quantification of Actin+ area for each coating type and stiffness. *N* = 3 cattle and *n* = 4 stiffnesses. Scale bars are 200 μm.

Due to differences in stiffness between the ECM array chip (10 kPa) and the differentiation assay conducted on plastic stiffness, as well as previous reports of FAPs responsiveness to substrate stiffness, FAPs were then differentiated on each coating at 2, 8, and 25 kPa for 10 days to determine if coatings impacted the mechanosensitivity of FAP cell fate (Loomis et al., 2022) (Figure 6a). White adipose tissue has approximately a stiffness of 2 kPa (Tharp et al., 2018), whereas 8 kPa closely matches the physiological stiffness of skeletal muscle (Fekete et al., 2018) as well as the stiffness of the ECM array chip, and 25 kPa reflects a fibrotic stiffness previously shown to drive higher ⍺SMA expression in murine FAPs (Loomis et al., 2022). We found that lipid‐accumulating area of bovine FAPs was affected by stiffness (*p* = 0.0336) but not coating (*p* = 0.6227), with a notable trend of increasing perilipin+ area/actin+ area on softer substrates (Figure 6b); myofibroblast area was not affected by stiffness (*p* = 0.2919) or coating (*p* = 0.3880) (Figure 6c). Additionally, we observed that FAPs preferentially attached to stiffer substrates, with nuclei count increasing with substrate stiffness (*p* = 0.0047) (Figure 6d). On softer substrates, FAPs clumped and formed self‐aggregates; this is reflected in the total actin+ area which increased with substrate stiffness until 25 kPa, but dropped off at TC plastic (*p* < 0.0001) (Figure 6e). Differentiation and accumulation of lipid droplets was still observed in these aggregates (Figure 6a). No significant differences in FAP end‐stage attachment were observed due to of coating (*p* = 4748 for nuclei count and *p* = 0.4366 for actin area) (Figure 6d,e). Overall, stiffness, but not coating, was a determinant of FAP differentiation into adipocytes, as well as their attachment during adipogenic differentiation in 2D culture.

## 4 DISCUSSION

Bovine FAPs are critical for skeletal muscle growth, repair, and meat quality through the generation of intramuscular adipocytes and ECM. For agricultural and cultivated meat interests, in vitro research with bovine FAPs provides an opportunity to capitalize on FAP cell behaviors to improve food quality influenced by fat and connective tissue. However, to achieve this, we require a deeper understanding of FAP cell behavior, as well as standardization and optimization of cell culture conditions. In other models, both the composition and stiffness of substrates have been shown to alter cell behavior (Hausman et al., 1996; Loomis et al., 2022; Shaheen et al., 2017; Takata et al., 2020; Vaz et al., 2012; Young et al., 2013). Despite the significance of the substrate and the recent interest in bovine FAPs, a thorough assessment of FAP responses to substrates is yet to be conducted, particularly in livestock‐derived cell lines. With an understanding of FAPs native location in the muscle's interstitial space and their interaction with the ECM, this paper aimed to assess a broad array of ECM protein coatings on bovine FAP behavior in vitro.

In existing literature, bovine cells are grown on various substrates such as TC plastic or coatings such as collagen 1 (Chung et al., 2021; Ding et al., 2018; Dohmen et al., 2022) and laminin (Stout et al., 2022; Stout et al., 2023). While our selected coatings did not differentially impact initial FAP attachment within 6 h, we did observe significant differences in 24‐h attachment due to coating. Notably, fibronectin coatings supported significantly more cells 24 h after plating, and we attribute this due to increased cell proliferation rates on fibronectin coatings. Both beneficial impacts of fibronectin on proliferation and cell yields were maintained when fibronectin was mixed with other ECM components, namely collagen VI. In the ECM, fibronectin assists in cell‐matrix adhesion through integrin binding (Parisi et al., 2020) and has demonstrated positive impacts for other cell types in attachment, proliferation, and differentiation (Arredondo et al., 2021; Vaz et al., 2012). Cell proliferation can be regulated through integrin binding by promoting expression of cyclins, proteins involved in regulating cell growth, and transitioning cells from the G1 phase to the synthesis phase of the cell cycle (Parisi et al., 2020). In the literature, there is evidence that integrin α5β1 adhesion to fibronectin can promote expression of cyclin D1, supporting cell progression through G1 (Danen & Yamada, 2001). For example, previous studies report fibronectin affecting cell proliferation in C2C12 cells (García et al., 1999), mammary epithelial cells (Williams et al., 2008), and bronchial epithelial cells (Han & Roman, 2006). Combined with existing literature on fibronectin and cell proliferation, our findings demonstrate this phenomenon for the first time in bovine FAPs and highlight fibronectin's potential to increase cell yields and proliferation rates.

While our original ECM screen indicated differences in FAP adipogenic differentiation across substrates, our more in‐depth assays with the selected four coatings did not corroborate that finding. Combined with the observed differences in attachment and proliferation, the shorter differentiation timeline necessary for use with the ECM array chip could be a factor in the inconsistencies moving to more rigorous testing. While collagen 6 has been shown to impact adipocyte behavior in other studies (Oh et al., 2021; Pasarica et al., 2009), we did not observe an effect on adipogenesis on collagen 6 coatings. Rather than substrate composition, we found that substrate stiffness and media type drove FAP adipogenic differentiation in vitro. FAPs had more lipid‐accumulating area in adipogenic media (BoFat) with or without FFA additions, while BoGrow media led to greater myofibroblast activation of FAPs. Notably, adipogenically differentiating FAPs occupied less total area than fibrogenically differentiating FAPs, and adipogenic FAPs had visible patches of unoccupied space where lipid‐accumulating cells likely detached. Detachment of adipocytes has been observed in 2D cell culture due to their increases in cell buoyancy associated with lipid accumulation (Dufau et al., 2021). While cell attachment was influenced by coating type, coatings did not dramatically impact cell fate compared to media type or substrate stiffness. Regarding adipogenic differentiation, in vivo intramuscular fat accumulation is dependent on a variety of factors including sex, breed, and age (Albrecht et al., 2006; Nguyen et al., 2021). In the present experiment, we observed this animal variation at the cellular level, and we have structured our data analysis to assess the change within an animal's response accordingly.

Within the same media type, we found FAP attachment was impacted by substrate stiffness. As substrate stiffness increased, so did the number of adhered FAPs. Additionally, the average area per cell increased with substrate stiffness through 25 kPa, but then decreased on TC plastic; this may be due to larger amounts of cells attached on TC plastic and thus less space available for each cell. Within adipogenic media, we found that FAPs had less relative lipid‐accumulating area as substrate stiffness increased. Specifically, bovine FAPs had the highest relative lipid‐accumulating area on our softest substrate, 2 kPa, which was chosen to represent the stiffness of white adipose tissue (Tharp et al., 2018); this aligns with existing literature on adipose stem cells, where adipogenic gene expression and lipid accumulation have been shown to increase on 2 kPa substrates (Young et al., 2013), and is the first study to support this phenomenon in bovine FAPs. Contrastingly, human and mouse‐derived FAPs have shown mechanosensitivity with regards to myofibroblast but not adipogenic differentiation with substrate stiffness (Loomis et al., 2022). While this was not observed in this study with bovine FAPs, only adipogenic differentiation was induced on varying stiffnesses for this cell fate's major importance to the cultivated meat and agricultural fields.

The present study further supports the use of MACS sorting for bovine FAPs (Guan et al., 2017) isolated from conventionally slaughtered bovine tissue. Combined with other recent work using FACS (Dohmen et al., 2022) and pre‐plating (Wang et al., 2023) to sort bovine FAPs, our work demonstrates methods and areas of optimization for in vitro bovine FAP protocols. As FAPs are the primary producers of both muscle ECM (Listrat et al., 2023) and marbling (Agricultural Marketing Service, n.d.) which drive meat quality and palatability, our present work presents in vitro methods to assess these factors from their cellular source for agricultural research (Molina et al., 2021). With new methods of meat production, such as the emerging field of cultivated meat, the present study emphasizes the important effect of substrate on cell behavior. For adherent culture in cultivated meat research, fibronectin could potentially be combined with artificial substrates and cell scaffolds to increase cell proliferation rates and more efficiently use limited stocks of cells. However, it is important to note that other ongoing research areas in cultivated meat, such as optimization of serum‐free media, may also impact cell attachment; thus, substrate composition may require further optimization under these conditions. Additionally, our finding of enhanced lipid accumulation on softer substrates and higher cell spreading on stiffnesses below TC plastic suggests softer substrates could be beneficial to maximize lipid production and minimize the number of cells needed to cover an area, though further characterization to fully elucidate the impact of substrate stiffness on bovine FAPs is necessary. Overall, the present work provides an extensive study of substrate conditions for FAP cell culture and highlights the importance of further investigation into bovine FAPs mechanosensitivity and relationship with their environment.

## AUTHOR CONTRIBUTIONS

P.G. and L.S.: Conceptualization; L.S.: Funding Acquisition; P.G., M.S., B.K., and N.M.: Investigation; P.G. and B.K.: Formal Analysis; P.G. and L.S.: Data Curation; P.G.: Visualization; P.G.: Writing original draft; P.G., M.S., B.K., N.M., P.V., and L.S.: Writing—review and editing; P.G., M.S., B.K., N.M., P.V., and L.S.: Approved final version of the manuscript.

## FUNDING INFORMATION

This study was funded by the National Science Foundation (2320899) and the National Institute of Health (R01AR079545).

## CONFLICT OF INTEREST STATEMENT

The authors declare no conflict of interest with the work presented.

## ETHICS STATEMENT

Animals were not covered under an experimental Institutional Animal Care and Use Committee (IACUC) protocol as tissue was collected based on availability post slaughter under USDA inspection at the UC Davis Meat Lab. Primary bovine cell use was approved under the Biological Use Authorization (BUA) #R2658 at the University of California, Davis.

## Supporting information


Data S1.

## Data Availability

Data can be made available by reasonable request to the corresponding author.
