# Supplementary material for: The impact of extracellular matrix proteins on bovine fibro‐adipogenic progenitor cell adhesion, proliferation, and differentiation in vitro
Source: Physiol Rep. 2025 May 1;13(9):e70283. doi: 10.14814/phy2.70283 (PMC12045701; doi:10.14814/phy2.70283)
Supplement: Supplementary file 1 — Data S1. [file PHY2-13-e70283-s001.pdf]

# Supplemental Figure 1

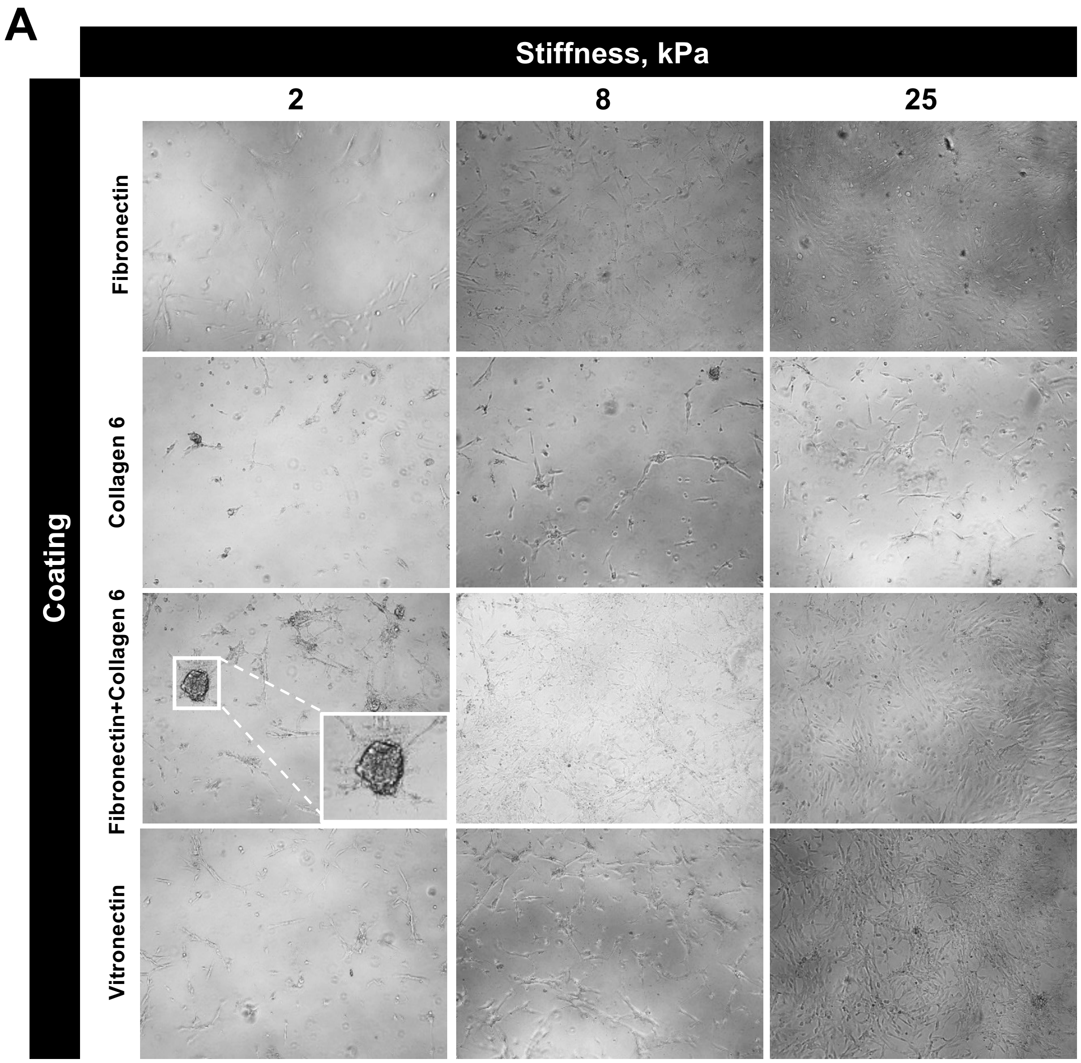


FAP 3-day attachment on selected coatings and stiffnesses. **(A)** Phase contrast live images of Animal 2 FAPs on 2, 8, and 25 kPa stiffnesses and each selected coating. Image is after 3 days of growth, with the same number and stock of FAPs plated on each condition.

# Supplemental Figure 2

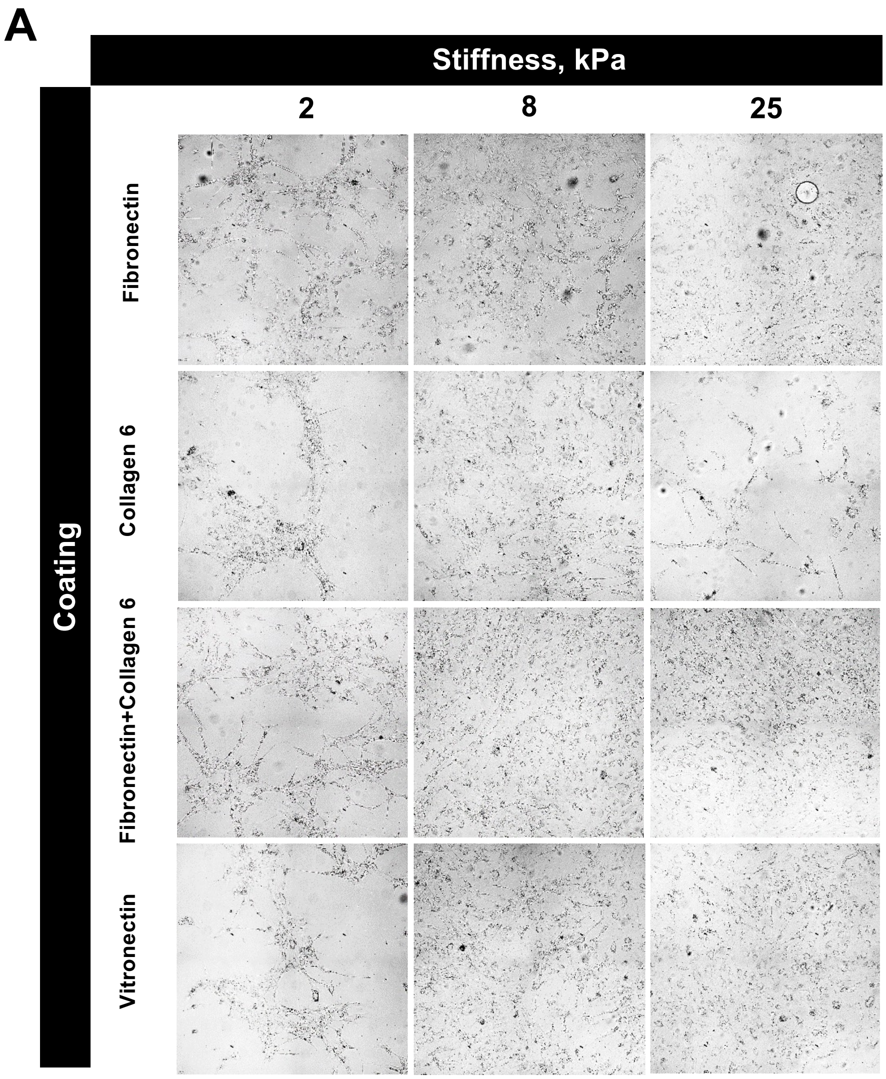


FAP at day 7 of adipogenic differentiation on selected coatings and stiffnesses. **(A)** Phase contrast live images of Animal 3 FAPs on 2, 8, and 25 kPa stiffnesses and each selected coating. Image is after 3 days of growth and 7 days of adipogenic differentiation in BoFAT media, with the same number and stock of FAPs plated on each condition.

## Supplemental Figure 3

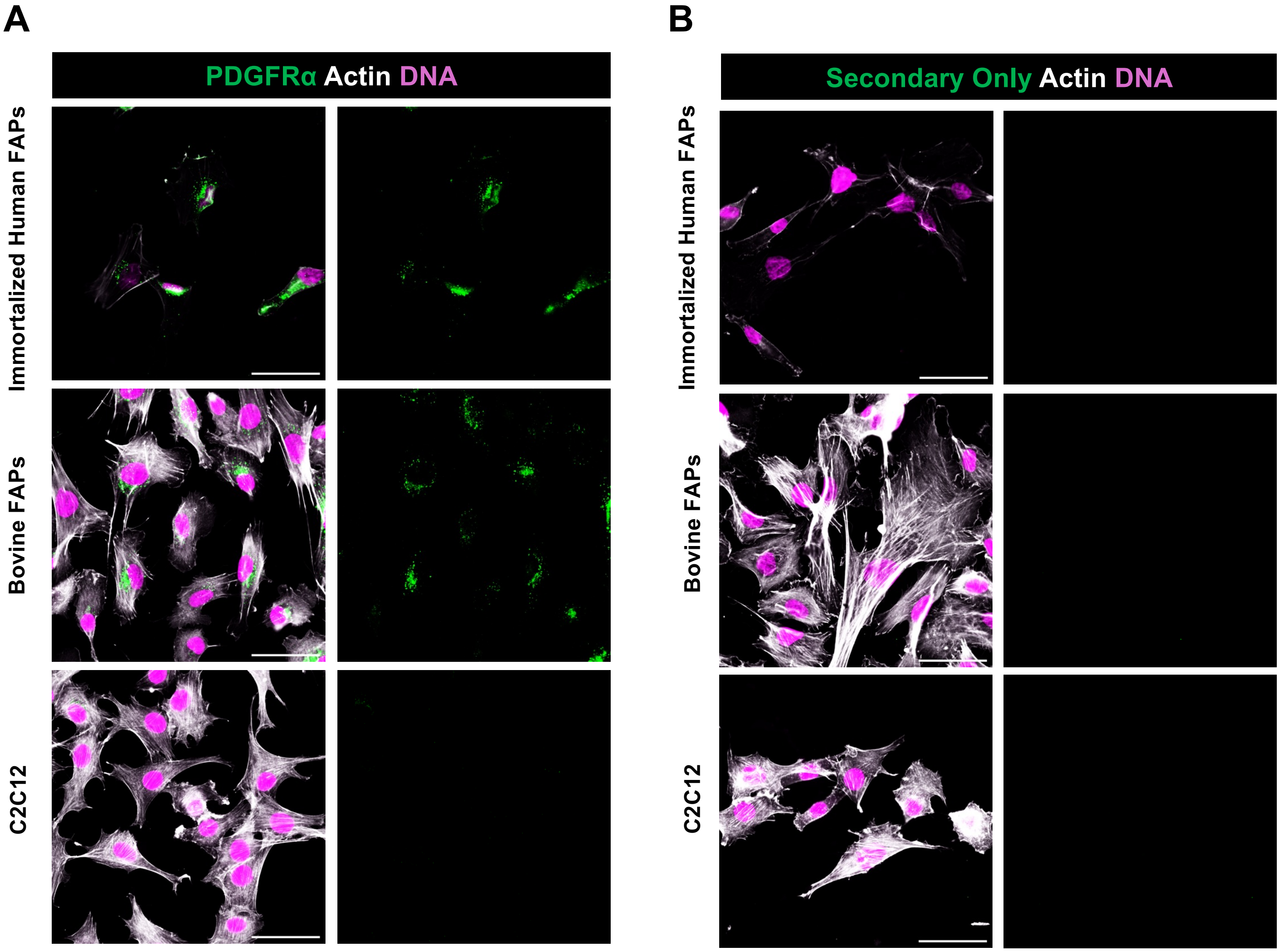


**PDGFRα expression. (A)** Immunofluorescent composite and PDGFRα single channel images in immortalized human FAPs (positive control), bovine FAPs, and C2C12 cells (negative control). Stained via Abcam 203491 at 1:75 dilution. **(B)** Immunofluorescent composite and PDGFRα single channel images for a secondary only control using 5% BSA in immortalized human FAPs, bovine FAPs, and C2C12 cells. Bovine FAPs are isolated from animal 1. Scale bars are 50μm.
